# The effect of mandibular flexure on the design of implant-supported fixed restorations of different facial types under two loading conditions by three-dimensional finite element analysis

**DOI:** 10.3389/fbioe.2022.928656

**Published:** 2022-08-29

**Authors:** Jing Gao, Xuejing Li, Jing He, Lulu Jiang, Baohong Zhao

**Affiliations:** ^1^ Center of Implantology School and Hospital of Stomatology, China Medical University, Liaoning Province Key Laboratory of Oral Diseases, Shenyang, China; ^2^ Shanghai Engineering Research Center of Tooth Restoration and Regeneration, Department of Prosthodontics, School and Hospital of Stomatology, Tongji University, Shanghai, China

**Keywords:** finite element analysis, mandibular flexure, implant-supported fixed restoration, stress distribution, facial type

## Abstract

**Objective:** Investigating the biomechanical effects of mandibular flexure (MF) on the design of implant-supported fixed restorations in edentulous jaws of different facial types.

**Methods:** Three-dimensional finite element models were established to analyze mandibular displacement and stress distribution of implant-supported fixed restorations (four or six implants, different implant numbers and sites, and the design of the superstructure across the dental arch in one or two or three pieces, under the loading conditions of maximum opening or right unilateral molar occlusion) in mandibular edentulous patients of three different facial types (brachyfacial, mesofacial, and dolichofacial types).

**Results:** The brachyfacial type presented higher mandibular flexure and stress in the overall restorative system, followed by the mesofacial and dolichofacial types. During jaw opening and occlusal movements, the one-piece framework showed the lowest bone stress values surrounding the anterior implants and gradually increased to the distal position, and the three-piece framework showed the highest stress values for peri-implant bones. Also, the split framework could greatly increase the stress on abutments and frameworks. Moreover, fixed implant prostheses with cantilevers can generate high amounts of biomechanical stress and strain on implants and surrounding bones. The bone surrounding the anterior implant increased in stress values as the most distal implants were more distally located regardless of frameworks. The zirconia framework demonstrated higher stresses than the titanium framework.

**Conclusion:** The design of edentulous fixed implant-supported restorations can be optimized for facial types. For patients of the brachyfacial type or with high masticatory muscle strength, the non-segmented framework without a cantilever provides an optimal biomechanical environment.

## Introduction

Dental implants have become a usual treatment in recent years for the edentulous mandible. But the predictiveness and long-term success of implant treatment are strongly influenced by the biomechanical environment. Displacement of the mandible is one of the main causes of posterior implant failure in implant restorations (Miyamoto et al., 2010). The “U-” or horseshoe-shaped mandible is used as a curved beam to carry bilateral and unilateral loads. As the mandible is pulled by the masticatory muscles attached to condyles, the elastic, anisotropic, nonhomogeneous tissue is deformed, and the width of the mandibular arch decreases from a few microns to 1 mm, with an average of 0.073 mm (Omar and Wise, 1981; English, 1993; Linkow and Ghalili, 1999; Shinkai et al., 2004; Carl, 2005). There are four types of mandibular flexure (MF) displacements: symphyseal bending associated with medial convergence, dorsoventral shear, corporal rotation, and anteroposterior shear (Abdel-Latif et al., 2000; Law et al., 2012; Sivaraman et al., 2016) which can create compressive, tensile, or shear stresses on the mandibular bone tissue, cause an accumulation of microdamage, and initiate bone resorption (Zaugg et al., 2012). In natural dentition, these adverse pressures caused by displacement can be protected during jaw movement, due to the presence of the periodontal ligament, which allows physiological movement of the teeth (Wenzel and Gröndahl, 1995; Borg and Gröndahl, 1996). On the contrary, in implant dentures, because of the absence of the periodontal ligament, the stresses act directly on the jawbone, leading to bone resorption. Meanwhile, for edentulous implant-fixed restoration, the stress concentration is easy to happen on the rigid framework, which splints multiple implants in a single unit (Hobkirk and Schwab, 1991; Varthis et al., 2019). Therefore, the effect of stresses caused by MF on both bone and restoration in edentulous implant-fixed restoration needs to be further studied.

Studies have shown that the value of MF is related to the facial type of the patient (Du Brul and Sicher, 1954; Osborne and Tomalin, 1964; Abdel-Latif et al., 2000; Chen et al., 2000).Three basic types of facial morphology exist: 1) brachyfacial type: low mandibular plane angle, short vertical facial height, and horizontal growth pattern; 2) mesofacial type: average mandibular plane angle, average vertical facial height, and average growth pattern; 3) dolichofacial type: high mandibular plane angle, long vertical facial height, and vertical growth pattern (Rickets et al., 1982; Abu Alhaija et al., 2010). The mesofacial type accounts for 70% of the population, and the other 30% were almost equally distributed as either brachyfacial or dolichofacial type (Sella Tunis et al., 2021). As the mechanical load increases, produced by masticatory muscles, not only does the mandible grow laterally with the increasing growth of bone seams, but it is also accompanied by an increase in the mandibular bending (Weijs and Hillen, 1986; van Spronsen et al., 1991; Serrao et al., 2003; Custodio et al., 2011). The stronger the masticatory muscles, the greater the MF. Since the brachyfacial type has the strongest masticatory muscles, it has the highest MF accordingly, followed by the mesofacial and dolichofacial types. To date, the study of the relationship between facial type and MF is still limited to natural dentition (Weinmann and Sicher, 1955). A comprehensive analysis of MF and its effect on the functional response to facial morphology has never been performed on the implant-supported prosthesis. Therefore, it is vital to clarify the influence of MF in different facial types on the implant denture and then provide clinical guidelines for the implant superstructure design.

For the design of implant-fixed dentures in the edentulous jaws, there are still a lot of controversies. Martin-fernandez et al. (2018) concluded that an integrated superstructure provides an optimal biomechanical environment under different loading conditions. However, others advocated that (Zarone et al., 2003) segmented superstructures did not restrict the physiological bending of the mandible, instead a more precise passive emplacement of the implant could be achieved. Thus, further clarification is needed. Therefore, the purpose of this study was to investigate mandible displacement and the stress distribution in fixed full-arch mandibular restorations with different designs of superstructures in three facial types through a three-dimensional finite element analysis (3D-FEA). Moreover, two modes of loading conditions, including opening and clenching, were compared.

## Materials and methods

### Mandible and framework model fabrication

**FIGURE 1:**
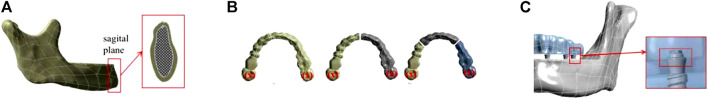
Three-dimensional finite element model. **(A)** Mandibular model; the ratio between cancellous and cortical bone thickness was approximately 10:1. **(B)** From left to right, one-piece, two-piece, and three-piece frameworks, respectively.**(C)** Implant system assembly.

Edentulous mandible geometry was obtained by 3D scanning of the human mandible with cone-beam CT-NEW TOM VGi (Danaher, USA) (Covani et al., 2011). The volume of the mandible was recreated using Mimics 10.0 software (Materialise, Belgium) and Geomagic Studio software (Rain Geomagic, United States) after filtering the 3D point cloud. Due to the effect of dental arch morphology on mandibular elastic bending was not statistically significant (Suresh, 2005), and the study unified and simplified the shape of the dental arch in the model design. A mandible of a mesofacial type was selected (Sella Tunis et al., 2021). Totally, the edentulous mandible possessed the following dimensions: symphyseal was 19 mm for inferosuperior height and 7 mm for buccolingual; the intercondylar distance was 96 mm; the intercoronoid distance was 92 mm; the arch width was 64 mm; and the mandibular angle to coronoid distance was 55 mm. The cancellous/cortical bone thickness is thought to be constant throughout the mandibular body, with a ratio of 10:1 (Figure 1A) (Zarone et al., 2003).

Framework’s geometry was defined by the dentition position, which was identified from the mandibular CT scan, and the teeth height was 8–13 mm. The model simulated a one-, two-, and three-piece superstructure. One-piece superstructure: splicing of six or four implants; two-piece superstructure: split at the mid-line (splicing of three implants each piece); and three-piece superstructure: divided into one anterior and two posterior sections (splicing of two implants each piece) (Figure 1B).

The implant of Nobel Parallel Conical Connection (Nobel Biocare, Sweden) (3.75/4.3 mm diameter, 10/18 mm height, and made of titanium alloy) was used as a reference for the modeling. Multi-unit Ti abutments (Nobel Biocare, Sweden) (3.5 mm height, 0°, or 30°angel) were patterned and screwed onto the implants for supporting the prosthetic framework. The “implant-multibase-screw” sub-structure is assumed to be a single unit: the friction and contact between the implant, abutment, and screw are not considered in this model (Favot et al., 2014).

### Material properties and interface conditions

All the materials used in models are assumed to be linearly elastic and homogeneous. The values of Young’s modulus and Poisson’s ratio are shown in Tables 1, 2 (Liao et al., 2008; Mazaro et al., 2011; Thompson et al., 2011). Final assembly between the mandible and the framework model was designed based on Boolean operations in UG10.0 software (Raindrop, United States), as shown in Figure 1C. The interface condition was set in Ansys Workbench 17.0 software (Dassault Systemes, France). The implant was regarded as achieving 100% osseointegration and perfect passive fit of the abutment and superstructure. These structures were set up to be fully bonded and free of any loosening.

**TABLE 1:** Mechanical properties of the materials of the mandible.

Category	Ex	Ey	Ez	Gxy	Gxz	Gyz	Vxyz	Vxz	Vyz
Cortical bone	12,500	17,900	26,600	4,500	5,300	7,100	0.18	0.31	0.28
Cancellous bone	210	1,148	1,148	68	68	434	0.055	0.055	0.322

E: Young’s modulus (MPa); G: Shear elastic modulus (MPa); V: Poisson’s ratio.

The direction of the elastic modulus of the bone cortex: X-radial; Y-tangential; Z-axial.

Direction of the cancellous bone elastic modulus: x-up and down; Y-near and far middle direction; Z-before and after.

**TABLE 2:** Mechanical properties of the materials of implant and superstructure.

Category	Young’s modulus (MPa)	Poisson’s ratio
Implant (titanium alloy)	114,000	0.35
Superstructure (zirconia)	186,000	0.28
Superstructure (titanium)	109,000	0.31

### Model meshing and design

**FIGURE 2:**
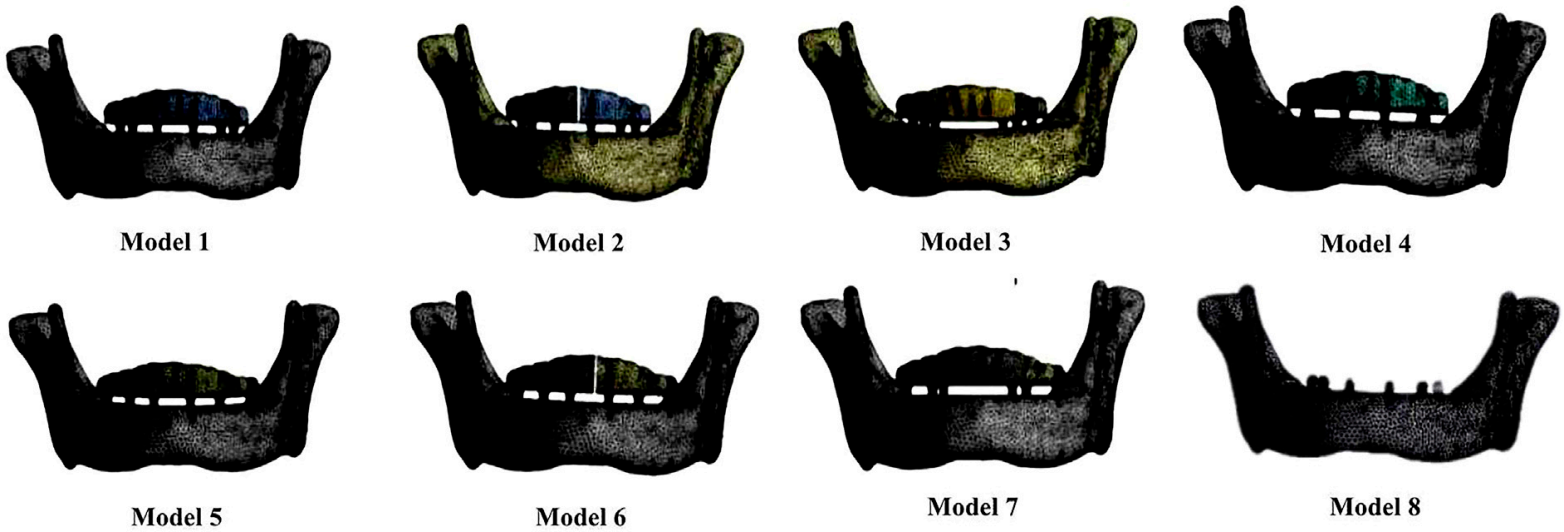
Model meshing and design.

Eight different 3D models were analyzed (Figure 2; Table 3). The tetrahedral mesh was used for the mandible and the prosthesis, which can refine the mesh of complex areas and meet the basic requirements of static analysis (Martin-Fernandez et al., 2018). The maximum mesh size and growth rate were 0.0018 m and 1.85, respectively. Elements and the number of nodes in the final 3D finite element models after mesh division are shown in Table 4.

·Model 1: the mandible model with six implants and a single cross-arch superstructure, restored to the bilateral first molars.
·Models 2: the mandible model with six implants and the superstructure divided into two freestanding sections at the symphyseal region, restored to the bilateral first molars.
·Model 3: the mandible model with six implants and the superstructure divided into three freestanding sections between the third and fourth teeth, restored to the bilateral first molars.
·Model 4: the mandible model with six implants and a single cross-arch superstructure, restored to the bilateral second molars.
·Model 5: the mandible model with six implants and the superstructure divided into two freestanding sections at the symphyseal region, restored to the bilateral second molars.
·Model 6: the mandible model with six implants and the superstructure divided into three freestanding sections between the third and fourth teeth, restored to the bilateral second molars.
·Model 7: the design of all-on-four: the mandible model with four implants and a single cross-arch superstructure, restored to the bilateral first molars. Two anterior straight implants were placed in the bilateral lateral incisor area, and two posterior implants tilted at a 45° angle to the occlusal plane were placed in the bilateral second premolar area with a cantilever of 13 mm.
·Model 8: the mandible model with six implants and without a framework (reference model).

**TABLE 3:** Model design.

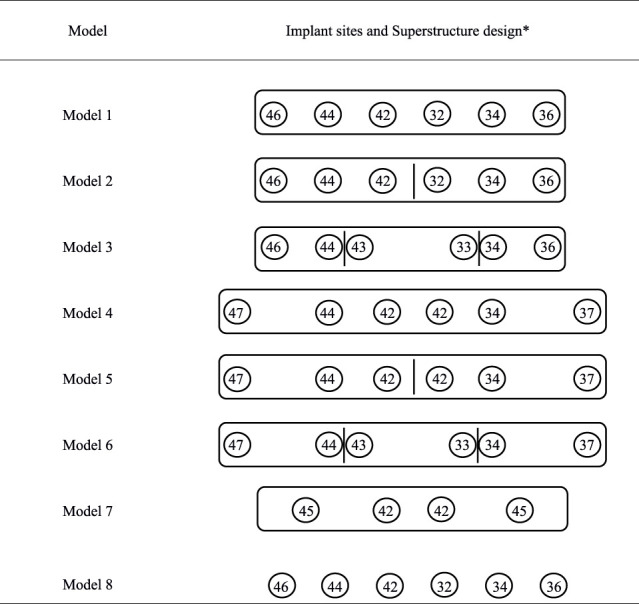

a The circular number represents the dental implant location. The frame represents the segmented design of the superstructure, that is, models 1 and 4 are one-stage superstructures; models 2 and 5 are two-stage superstructures; models 3 and 6 are three-stage superstructures; model 7 is designed for all-on-four; model 8 has no superstructure for reference. The sites of implants were placed 12 mm (32, 42), 21.2 mm (33, 43), 32.7 mm (34, 44), 45.6 mm (35, 45), 60 mm (36, 46), and 77.5 mm (37, 47) from the midline, respectively, on each side.

**TABLE 4:** Number of elements and nodes of the 3D finite element models.

	M1	M2	M3	M4	M5	M6	M7	M8
Nodes	519,317	507,300	507,974	532,847	521,788	522,804	498,536	423,591
Elements	360,964	352,125	352,595	369,458	361,307	361,988	346,111	296,010

### Classification and setting of facial types and genders

**FIGURE 3:**
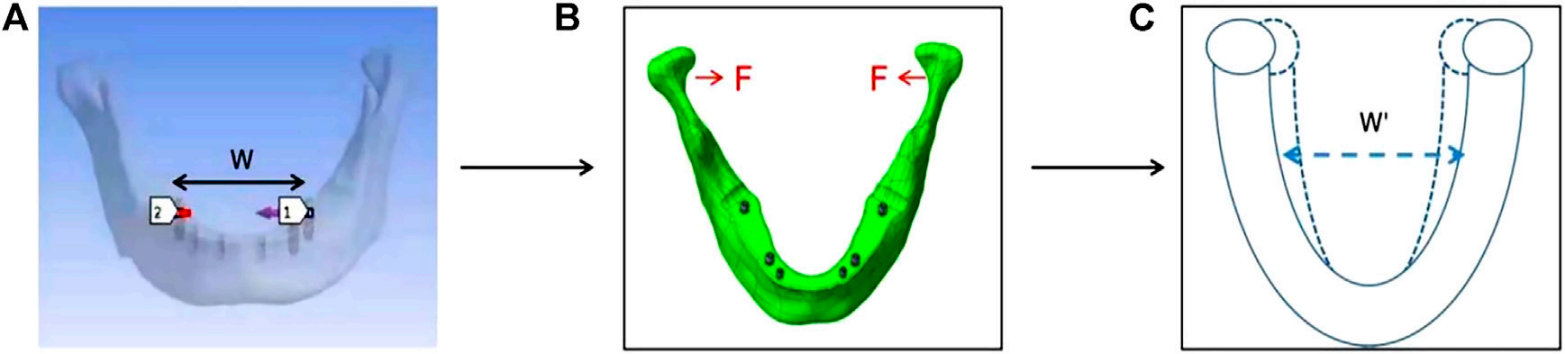
Method of deriving loading force values, taking the brachyfacial type/male as an example. **(A)** Set the horizontal path *p* between the bilateral first molars (between 1 and 2 in Panel 3A) to measure the arch width. **(B)** Apply the cohesive force **(F)** at the bilateral condyles. **(C)** Adjust the value of F and measure the amount of displacement between the bilateral first molars (△W); when ΔW equals 1.09 mm (X), the corresponding F value (41 N) was equivalent to the muscle strength of brachyfacial type/male.

During maximum opening, the mean value of MF (X) at the first molar varied widely by facial types and genders, mainly depending on the strength of the muscles attached to the bilateral condyles (Costa et al., 2019). In FEA, the cohesive force (F) applied to the bilateral condyles was used to mimic the muscle strength (Figure 3). In this study, based on X (Table 5) reported by Prasad et al. (2013), the F value for three facial types (brachiofacial, mesofacial, and dolichofacial types) and two genders (male and female) was calculated on model 8 (the reference model), as follows: as the F value increases, the change in the arch width (ΔW) between the bilateral first molars was recorded. When ΔW equals X, the corresponding F value was determined, which was equivalent to the muscle strength of each facial type or gender. Finally, six sets of F-values, corresponding to three facial types or two genders, were obtained.

**TABLE 5:** Mean cohesion displacement of bilateral first molars with wide mouth opening in different facial types and genders.

Facial type/gender	Displacement (X:mm) (Prasad et al., 2013)
Brachyfacial type/male	1.09
Brachyfacial type/female	1.15
Mesofacial type/male	0.62
Mesofacial type/female	0.76
Dolichofacial type/male	0.43
Dolichofacial type/female	0.36

### Loading conditions

Both conditions were performed on seven models (Figure 3).

**FIGURE 4:**
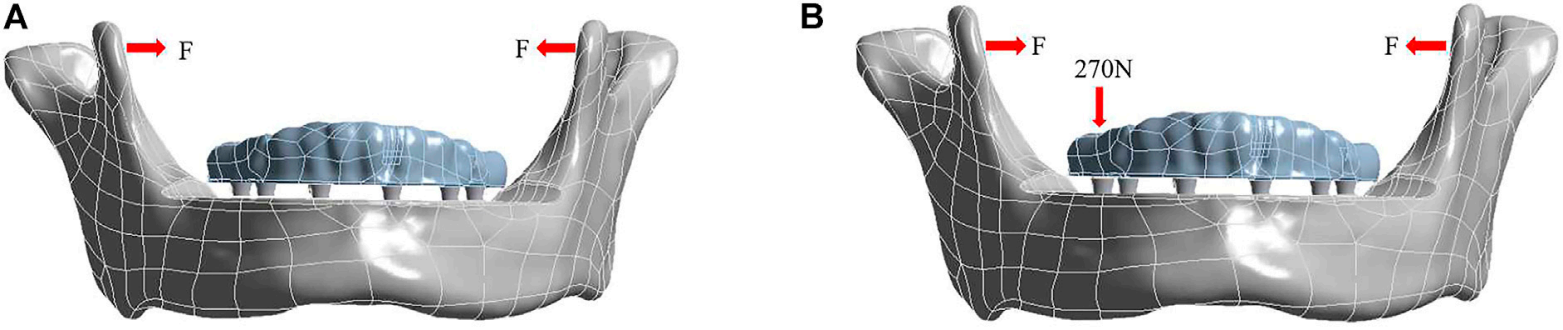
Loading conditions under the condition of maximum opening of the jaw **(A)** and right unilateral molar clenching **(B)**.

·Maximum opening of the jaw (Figure 4A). Various F-values obtained from the section “Classification and setting of facial types and genders” were applied to each model (three facial types and two genders were analyzed.)

·Right unilateral molar clenching (Figure 4B). A vertical bite force of 270 N (ElSyad et al., 2019) was applied on the central fossa of the right first molar and F = 10 N (the group of the mesofacial type/male was chosen to be assessed).

### Stress visualization analysis

The von Mises stress values were used for stress analysis, and the unique stress components contained in all eight models were summarized. The von Mises stress value is defined as follows (Zarone et al., 2003):

$$\text{σ}\text{=}\sqrt{\frac{1}{2}\left[{\left({\text{σ}}_{1}-{\text{σ}}_{2}\right)}^{2}+{\left({\text{σ}}_{2}-{\text{σ}}_{3}\right)}^{2}+{\left({\text{σ}}_{3}-{\text{σ}}_{1}\right)}^{2}\right]}.$$

The stress visualization is depicted by false colors on the geometric model: dark blue areas mean unstressed areas, while red areas mean more stressed areas.

## Results

Various F-values were obtained from the “Classification and setting of facial types and genders” section for different face types and genders as shown in Table 6. We evaluated mandibular displacement and stress distribution in both the mandible and denture of different patient characteristics (facial types and genders), prosthesis design (framework design, implant sites and number, and superstructure materials), and two different loading conditions.

**TABLE 6:** Cohesion force at the bilateral condyles in different facial types and genders.

Facial type/gender	Applied load (N)
Brachyfacial type/male	41.0
Brachyfacial type/female	43.5
Mesofacial type/male	23.5
Mesofacial type/female	28.5
Dolichofacial type/male	16.5
Dolichofacial type/female	13.5

### Mandibular displacement

#### Patient characteristics

As shown in the top (titanium-maximum opening) columns of Table 7, within different facial types, the amount of mandibular displacement was the largest in the brachyfacial type, followed by mesofacial and dolichofacial types, but all groups represented the same displacement pattern. For gender, mandibular displacement in the female group was slightly higher than that in the male group of brachyfacial and mesofacial types but lower in the dolichofacial type. Because the mesofacial type was the most prevalent (70%) in the population (Sella Tunis et al., 2021), the group of the mesofacial type (male) was chosen to assess the effect of other factors.

**TABLE 7:** Mandibular displacement at the bilateral first molar level in different facial types and genders in the mandibular opening and clenching (male/female).

	Model	Brachyfacial type (μm)	Mesofacial type (μm)	Dolichofacial type (μm)	Relative displacement
Titanium-maximum opening	Model 1	508.5/540	291.5/353.5	198.5/167.6	0.47
	Model 2	920/976	527/640	359/303	0.84
	Model 3	954/1,012	547/663	372.5/314	0.87
	Model 4	513/544	294/356.5	200/169	0.47
	Model 5	933/989	535/648	364/307	0.85
	Model 6	956/1,015	548/664.5	373/314.5	0.88
	Model 7	618/655	354/430	241/203.5	0.57
	Model 8	1,091/1,150	625/760	426/360	1^b^
Zirconia^a^-maximum opening	Model 1		270.4/─		0.43
	Model 2		522/─		0.83
	Model 3		543.9/─		0.87
	Model 4		272.6/─		0.43
	Model 5		528.8/─		0.85
	Model 6		544.7/─		0.87
	Model 7		336/─		0.53
	Model 8		625/─		1^b^
Titanium-right unilateral molar clenching^a^	Model 1		57.2/─		0.23
	Model 2		227.5/─		0.92
	Model 3		215.9/─		0.87
	Model 4		48.5/─		0.20
	Model 5		215.55/─		0.87
	Model 6		210.77/─		0.85
	Model 7		66.8/─		0.27
	Model 8		247/─		1^b^

a Since all three facial types represented the same pattern of displacement and the mesofacial type was the most prevalent in the population (70%) [Sella Tunis T, et al., 2021], the mesofacial-type (female) group was chosen to assess the effects of other factors.

b The relative displacement between the bilateral first molars of model 8 was set to 1, to compare the effect of other designs on the amount of jaw displacement.

#### Prosthesis design

In the study, the relative displacement between the bilateral first molars of model 8 was set to 1, to compare the effect of other designs on the amount of jaw displacement. During the maximum opening, minimum flexibility of the mandible was detected in models 1, 4, and 7 (full-arch bridge with or without cantilever), relative displacements of which were 0.47, 0.47, and 0.57, respectively (at the bilateral first molar region in the condylar convergence direction). For the full-arch bridge, the flexibility increased as the number of implants decreased (models 1 and 4 vs. model 7). A higher level of flexibility (0.85) was detected in models 2, 3, 5, and 6 (segmental framework). Therefore, compared with full-arch bridges, segmented superstructures favor in preserving the physiological bending of the mandible. Regarding the superstructure material, the mandible displacement of the titanium group was slightly higher than that of the zirconia group of the one-piece framework, with no significant difference in the segmental framework, as shown in the middle (zirconium-maximum opening) columns of Table 7.

#### Loading condition

As shown in the bottom (titanium-unilateral molar clenching) columns of Table 7, in right molar occlusion, the effect of the superstructure design on MF was more pronounced than that in the opening. The relative displacements of the one-piece framework were only 0.20–0.27 (models 1, 4, and 7), while relative displacements of segmented frameworks (models 2, 3, 5, and 6) were more than 0.85.

### Stress distribution (von Mises stresses) in the mandible

#### Patient characteristics

**FIGURE 5:**
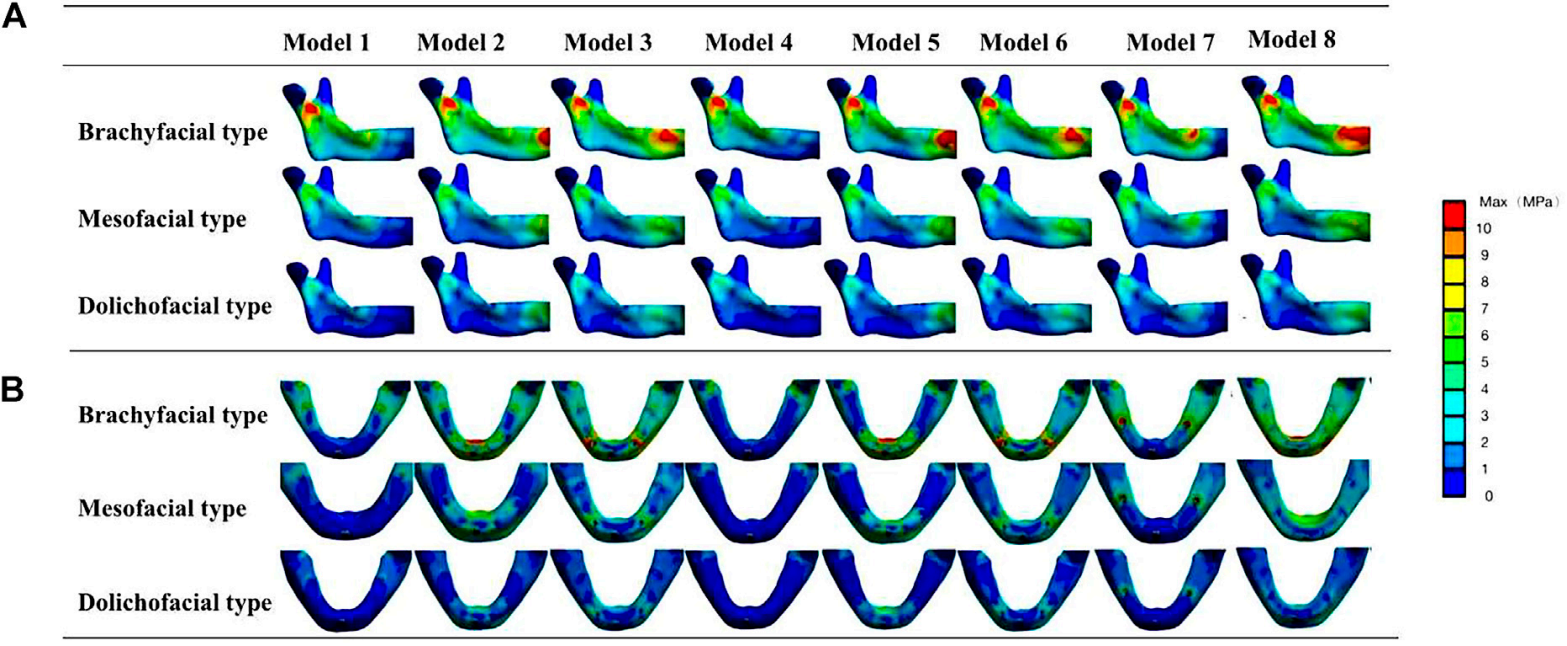
Von Mises stresses in the mandible of different facial types. **(A)** Inside view of the mandibular ascending ramus. **(B)** Occlusal view of the mandibular body. The stress peak values of the mandible mainly appear at the mandibular median joint, below the sigmoid notch of the mandibular ascending ramus and at the bone interface of the implants.

As shown in Figure 5, in three facial types, the stress in the mandible was the highest in the brachyfacial type, followed by mesofacial and dolichofacial types, and all groups represented the same pattern of stress distribution.

For gender, as shown in Table 8, the maximum von Mises stress of the mandible in the female group was slightly higher than that in the male group of brachyfacial and mesofacial types but lower for the dolichofacial type. Both patterns of stress distribution were the same. Because the mesofacial type was the most prevalent (70%) in the population (Sella Tunis et al., 2021), the group of the mesofacial type (male) was chosen to assess the effect of other factors.

**TABLE 8:** Maximum von Mises stresses on the mandible and prostheses in different facial types and genders in the mandibular opening (male/female) (MPa).

	Model	Distal implant	Middle implant	Mesial implant	Superstructure	Mandible
Brachyfacial type	Model 1	171/181	62/65	67/72	171/182	181/192
	Model 2	150/161	82/87	348/369	455/483	197/209
	Model 3	95/102	186/198	412/438	509/540	182/193
	Model 4	129/138	55/58	70/74	195/207	179/190
	Model 5	125/152	87/92	399/423	484/513	156/166
	Model 6	117/124	237/251	419/445	555/590	188/200
	Model 7	191/202	─	106/111	214/227	144/153
	Model 8	48/49	46/49	64/66	─	158/168
Mesofacial type	Model 1	104/113	40/42	41/45	98/105	103/110
	Model 2	86/104	47/56	213/224	261/316	113/126
	Model 3	55/67	111/135	265/322	292/354	105/137
	Model 4	77/86	36/44	40/50	112/136	103/127
	Model 5	75/87	50/61	229/278	277/336	90/125
	Model 6	67/83	142/172	270/326	320/386	107/109
	Model 7	112/135	─	66/80	122/149	83/131
	Model 8	29/35	26/33	36/43	─	91/100
Dolichofacial type	Model 1	64/56	25/20	27/22	69/56	73/60
	Model 2	60/49	33/27	140/114	183/150	79/65
	Model 3	38/31	75/60	166/136	197/168	72/60
	Model 4	52/43	28/23	29/23	79/54	72/59
	Model 5	50/41	35/28	160/131	195/159	63/51
	Model 6	47/38	95/78	169/138	223/183	76/62
	Model 7	77/67	─	42/35	86/70	58/47
	Model 8	27/15	19/15	25/16	─	64/52

#### Prosthesis design

**FIGURE 6:**
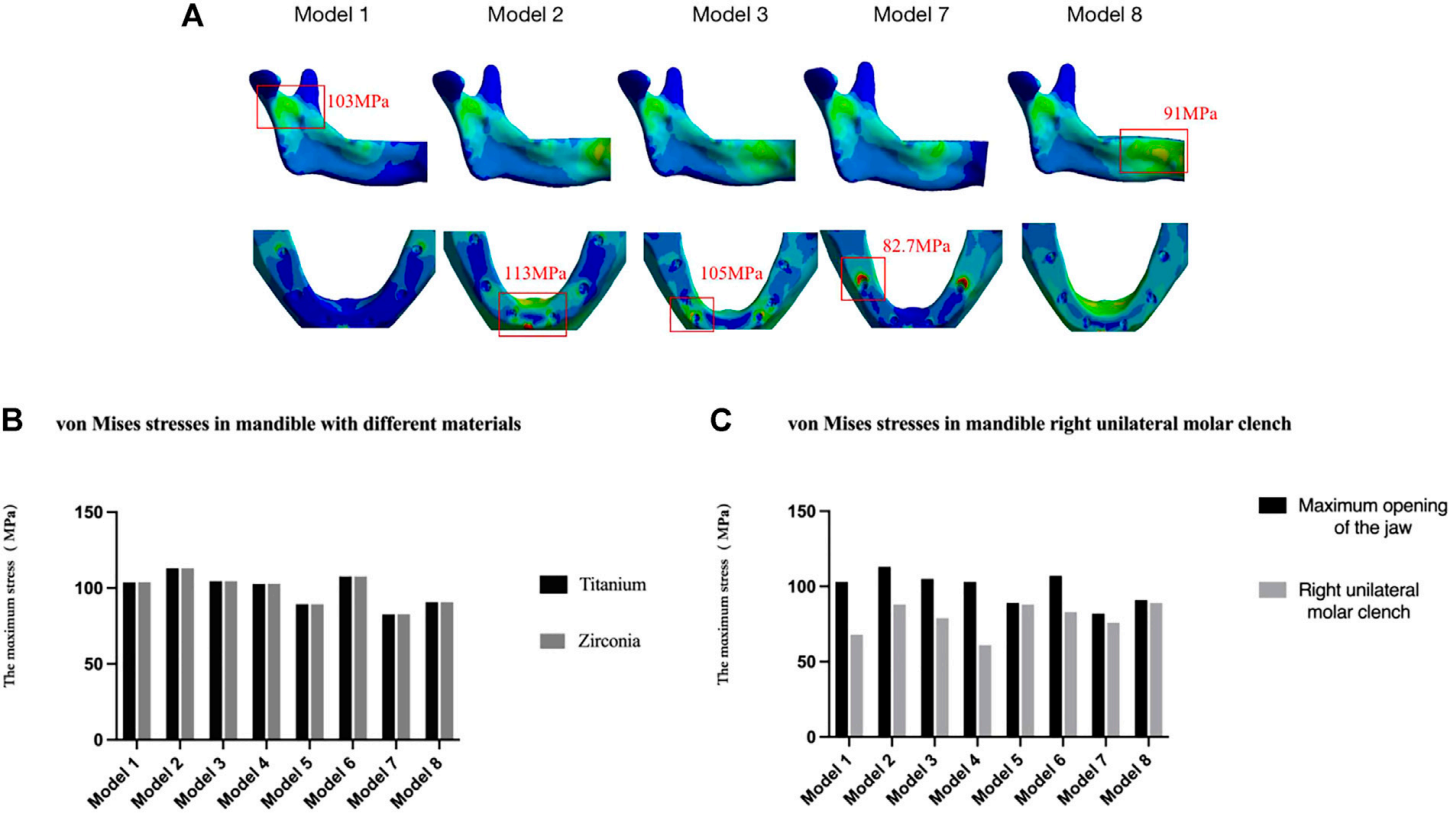
Von Mises stresses in the mandible of different denture designs. **(A)** Inside view of the mandibular ascending ramus and occlusal view of the mandibular body in different segmental frameworks. Only pseudo-color images of stress distribution for models 1, 2, 3, 7, and 8 are shown in Figure 6A due to the distribution of the stress in the jaws is the same for the same segmental frameworks (model 1 vs. 4, model 2 vs. 5, model 3 vs. 6). **(B)** Different framework materials and **(C)** right unilateral molar clenching.

The stress distribution at the mandible level during maximum opening loading with or without a framework is shown in Figure 6A, and a quantitative analysis was performed (Table 8). According to Figure 5, the distribution of the stress in the jaws is the same for the same segmental frameworks (model 1 vs. 4, model 2 vs. 5, and model 3 vs. 6). Therefore, only pseudo-color images of stress distribution for models 1, 2, 3, 7, and 8 are shown in Figure 6A. In model 8 (without superstructure), the stress on the mandible was mainly focused at the symphyseal and below the sigmoid notch of the ascending ramus (91 MPa). In the model with the full-arch design without a cantilever (models 1 and 4), only a small amount of stress was detected below the sigmoid notch of the ascending ramus (103 MPa). In the model with a cantilever (model 7), the bone interface around the distal tilted implant was subjected to greater stress (82.7 MPa), which probably increased the risk of bone resorption around implants (Barbier and Schepers, 1997). In models with the segmented framework (models 2, 3, 5, and 6), the stress peaks occurred mainly below the sigmoid notch of the ascending ramus and at the peri-implant bone interface (113, 105, 90, and 107 MPa in models 2, 3, 5, and 6, respectively). The stresses in the peri-implant alveolar bone were more significant in the three-stage framework. Regarding the superstructure material, there was no difference in the stress distribution of the mandible between titanium and zirconia groups. (Figure 6B).

#### Loading condition

During the right unilateral molar clenching, lower stress was observed in the mandible of all types of frameworks, compared to opening movements (Figure 6C). The stress on the jawbone is greater in segmental frameworks (models 2, 3, 5, and 6) than in the one-piece framework (models 1, 4, and 7.). According to the results, although inhibiting the physiological bending of the mandible, a full-arch superstructure without a cantilever restored the bone stress distribution observed in the restorative mandible, regardless of movement modes.

### Stress distribution (von Mises stresses) in prostheses

#### Patient characteristics

**FIGURE 7:**
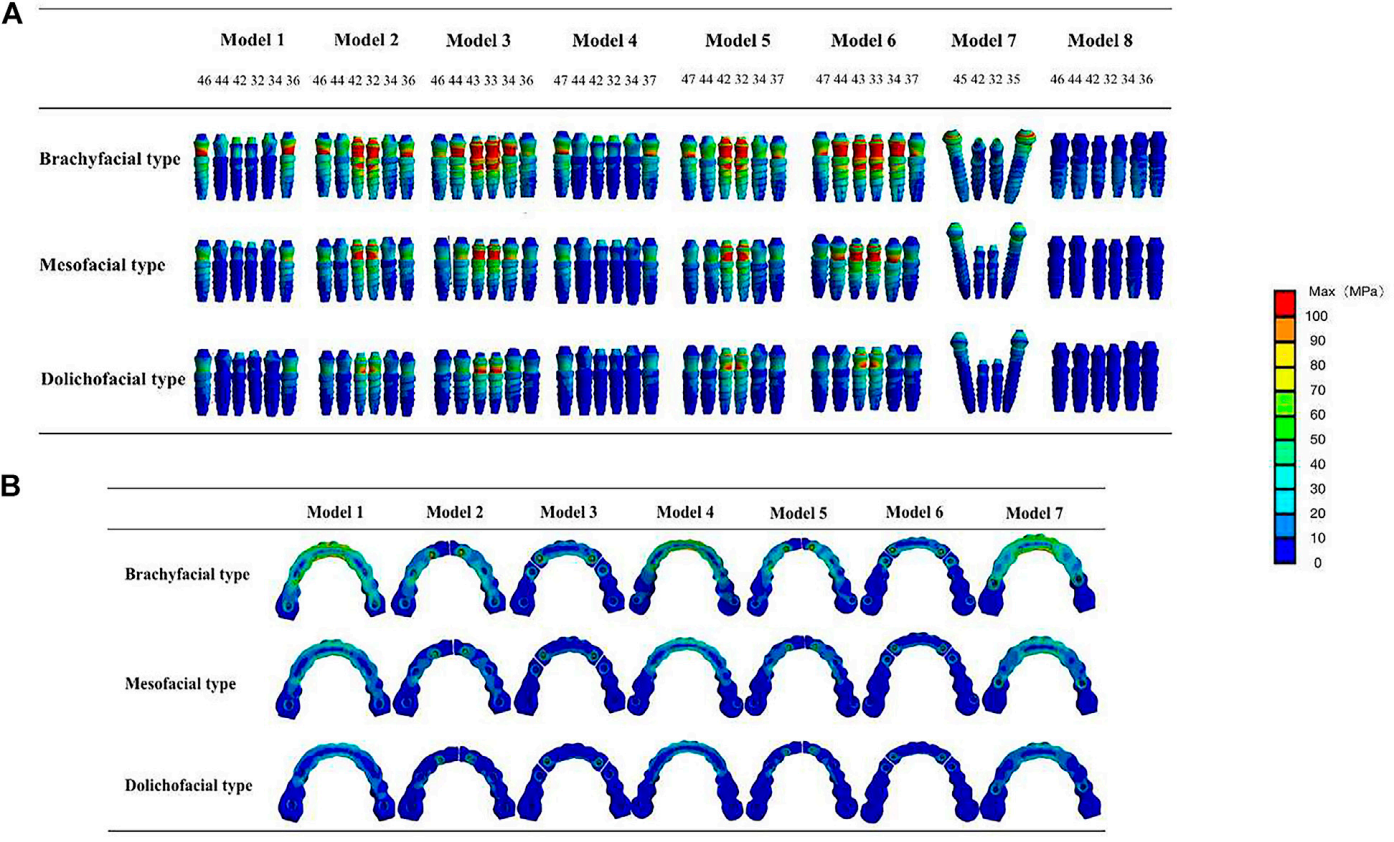
Von Mises stresses in prostheses of different facial types. **(A)** Von Mises stresses in implants. **(B)** Von Mises stresses in the framework. The stresses in prostheses were the largest in the brachiofacial type, followed by mesofacial and dolichofacial types, but all groups represented the same displacement pattern.

Within different facial types, the stress in the prostheses was the highest in the brachyfacial type, followed by mesofacial and dolichofacial types, but all groups represented the same pattern of stress distribution (Figure 7). For gender, the maximum von Mises stress in prostheses in the female group was slightly higher than that in the male group of brachyfacial and mesofacial types but lower in the dolichofacial type (Table 8). Because the mesofacial type was the most prevalent (70%) in the population (Sella Tunis et al., 2021), the group of the mesofacial type (male) was chosen to assess the effect of other factors.

#### Segmental design of the framework

**FIGURE 8:**
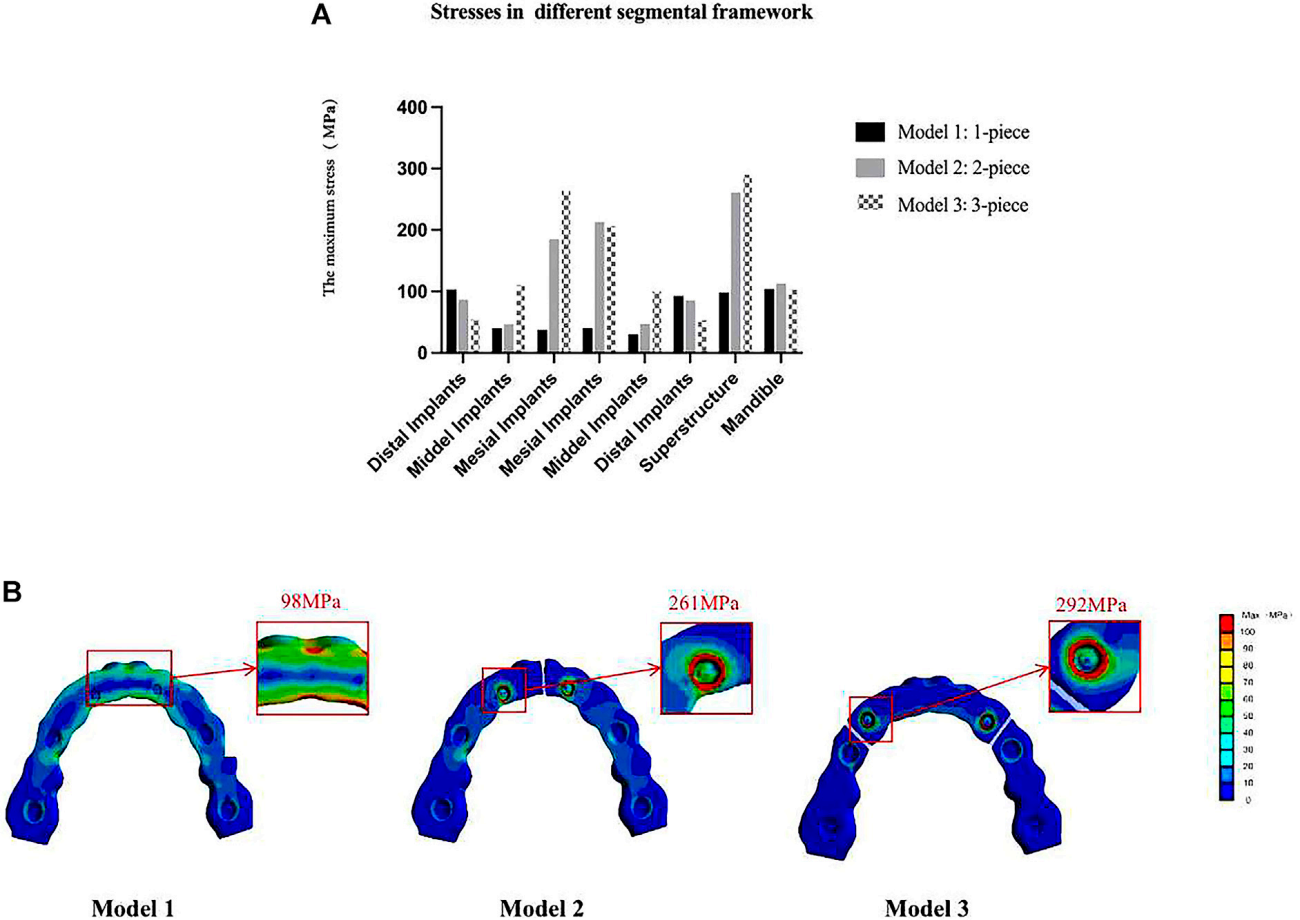
Von Mises maximum stress values **(A)** and pseudo-color stress distribution **(B)** in different segmental prosthetic frameworks of the mesofacial type/male. In the one-piece framework prosthesis, the largest bone stress occurred around distal implants and the mid-line of the superstructure. In the two-piece and three-piece framework prostheses, stress was mostly significantly concentrated at the joint between the abutments and superstructure (42–32, 33–34, and 43–44).

As shown in Table 8; Figure 8A, during mandibular maximum opening movements, in the one-piece framework restorations, the largest stress occurred around distal implants on both sides (104 and 77 MPa in models 1 and 4, respectively), progressively decreasing toward more mesial positions. In the two-piece framework restorations, the maximum stress was observed surrounding the lateral incisor location (213 and 219 MPa in models 2 and 5, respectively). The three-piece framework restorations, in which the maximum stress was observed surrounding the canine location (265 and 270 MPa in models 2 and 5, respectively), showed greater stress than one-piece and two-piece frameworks.

At the level of the framework, in the models with a full-arch framework (models 1, 4, and 7, Figure 8B), a larger stress concentration was detected on the mid-line of the superstructure (98 and 112 MPa in models 1 and 4, respectively). Also, the stress detected at the mid-line of the superstructure was reduced by splitting the latter into two or three freestanding portions. But, in models 2, 3, 5, and 6, with the segmental framework, stress was mostly significantly concentrated at the joint between the abutments and superstructure (261, 292, 277, and 320 MPa in models 2, 3, 5, and 6, respectively), which would increase the significant incidence of mechanical problems. Therefore, a one-piece framework may be more conducive to stress distribution in the framework.

#### The number and site of implant

**FIGURE 9:**
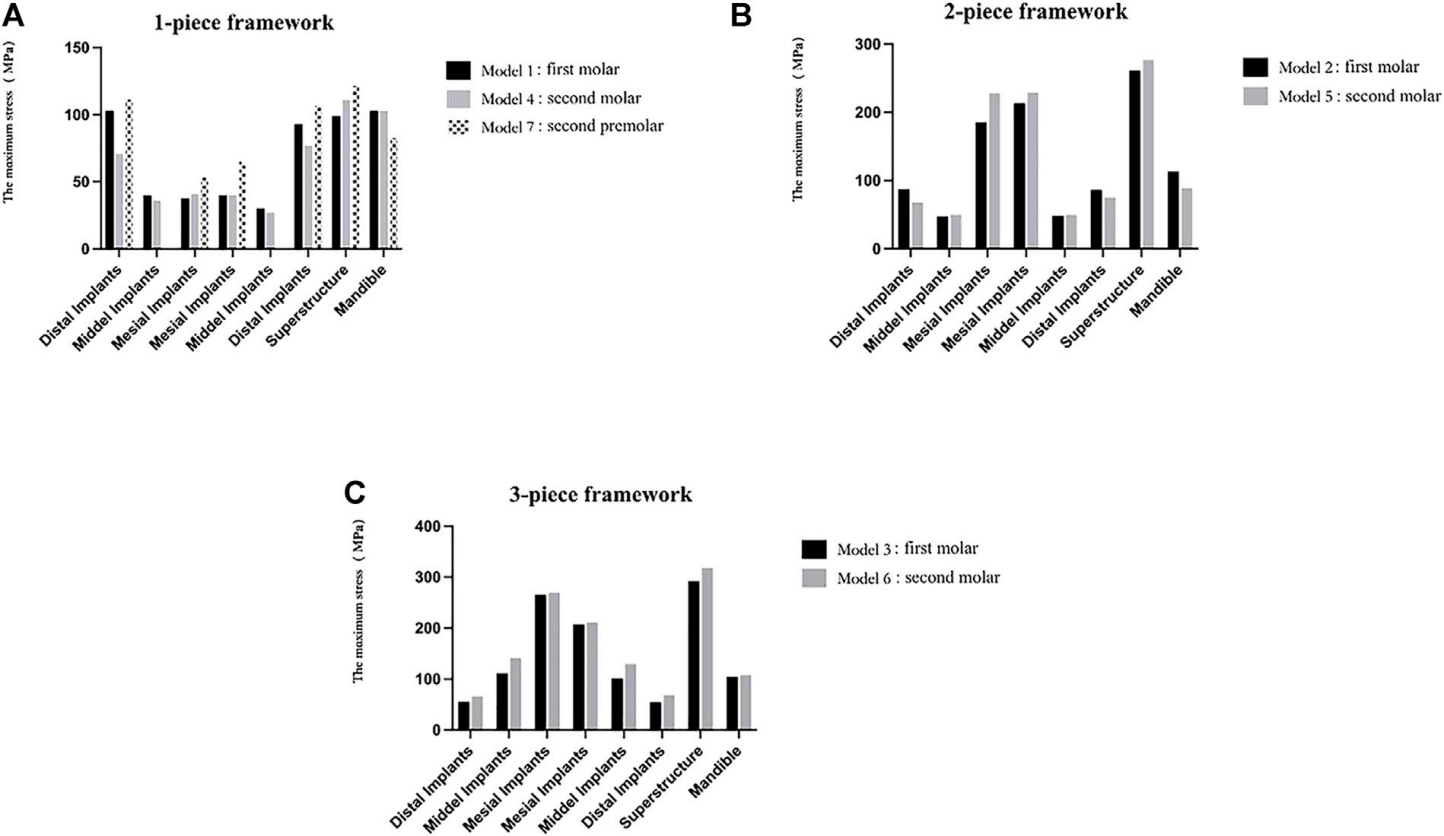
Maximum von Mises stresses on the mandible and prostheses with different numbers and sites of implants. In the one-piece superstructure **(A)**, the stress on the prosthesis in model 7 (with cantilever) is the largest; in one- **(A)** or two-piece frameworks **(B)**, the stress on the mesial implants and the superstructures increased as the distal implants were more distally located; in the three-piece framework **(C)**, stress values of the mandible, prostheses, and any implants increased as the distal implants were more distally located.

As shown in Figure 9A, in a one-piece framework with four implants (model 7, with a cantilever), the stress on all components of the restoration was greater than in a one-piece frame with six implants (models 1 and 4, without a cantilever), that is, the more the implants of the denture, the lower the von Mises stress on the denture.

From the stress distribution in the restorative typologies reported in Figures 9A–C, it can be noted that, in one- (comparing models 1 and 4) or two-piece frameworks (comparing models 2 and 5), the more distally the distal implant is located, the higher the stresses at the mesial implants (32, 42) and the superstructure will be. Also, in the three-piece framework (comparing models 3 and 6), stress values of the framework and any implants increased as the distal implants were more distally located.

#### The material of the framework

**FIGURE 10:**
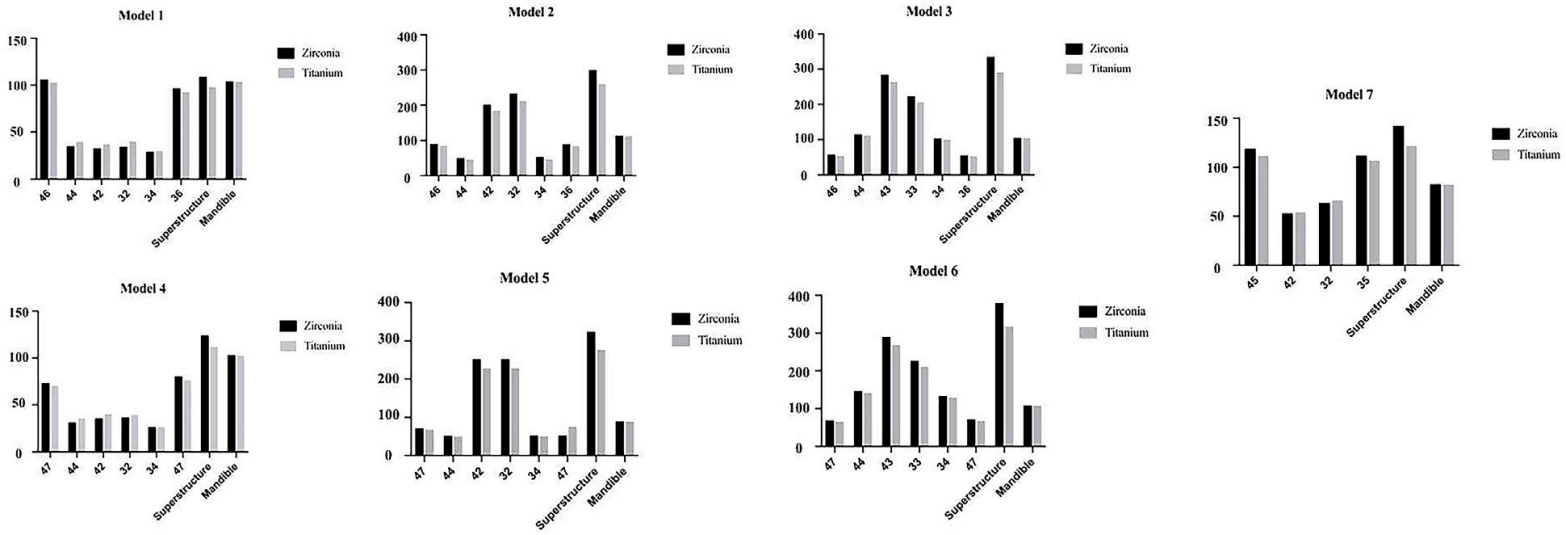
Maximum von Mises stresses on mandible and prostheses with different materials in mesofacial type/male.

As shown in Figure 10, according to the results, in the titanium group, the posterior implants (35, 36, 45, 46) and restorations in the one-piece framework and all components in the segmented framework were under less stress than in the zirconia group, except for the anterior implants (32, 34, 44, 42) in the one-piece framework (models 1, 4, 7), which were under greater stress. Therefore, titanium is more recommended as the superstructure material for the mandibular edentulous implant-supported fixed denture.

#### Loading condition

**FIGURE 11:**
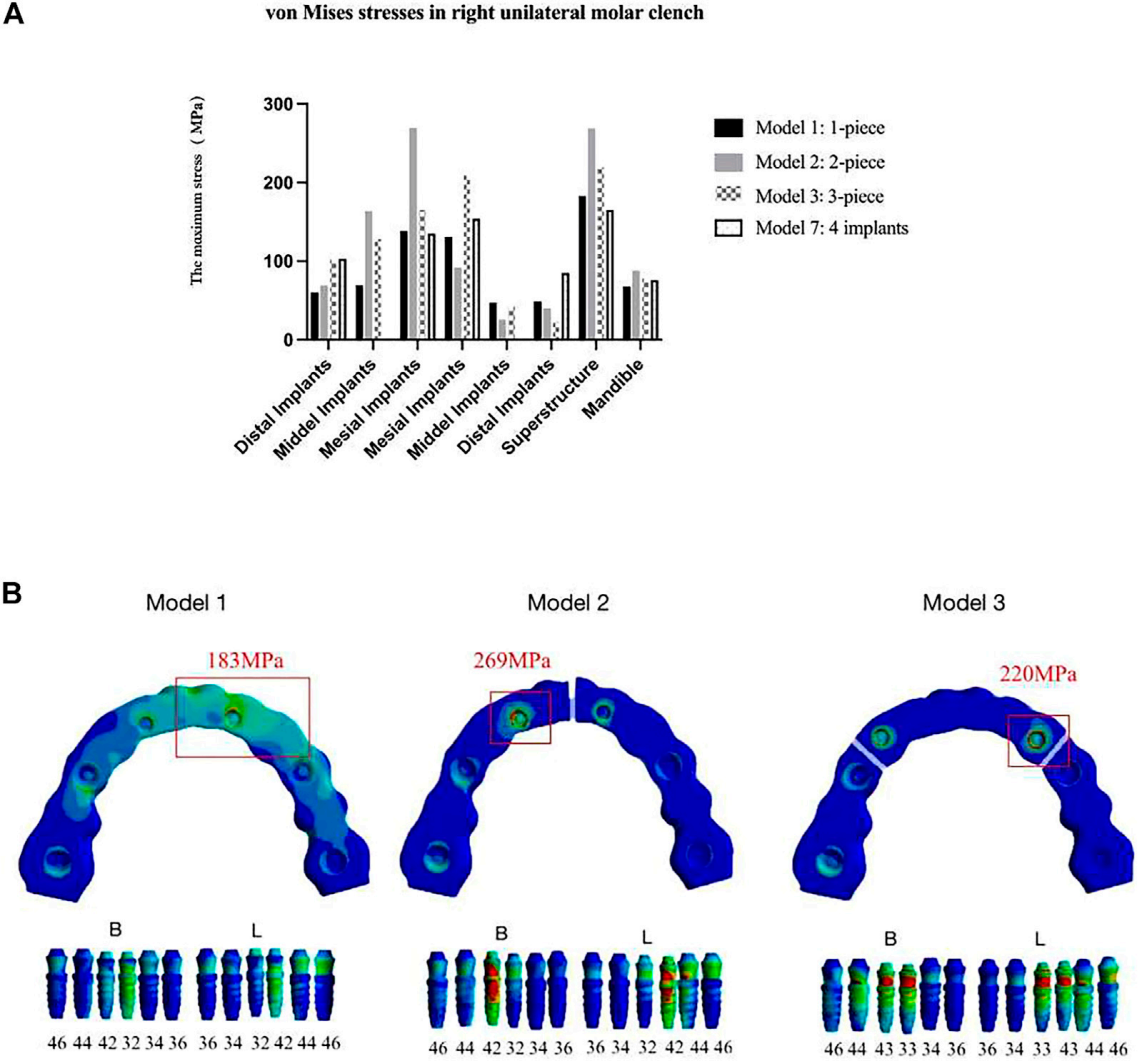
Von Mises maximum stress values **(A)** and pseudo-colored stress distribution **(B)** in the right unilateral molar clenching. The largest stress values were all recorded around mesial implants in seven models during right molar clenching.

During the right unilateral molar clenching, the stress distribution of the superstructure is the same as that of the maximum opening. The stress was concentrated at the midline area of the full-arch framework and connection between the abutment and superstructure of the segmental framework (Figure 11) but not as uniform as stresses during the opening. The maximum von Mises stresses on the implants in view of buccal and lingual sides were not exactly the same (Nokar and Baghai Naini, 2010; Law et al., 2014), as shown in Table 9. Regardless of the type of the framework, the maximum stress was all concentrated around anterior implants. In contrast, the segmented framework provided the worse biomechanical environment, and the two-piece framework produced the highest values of stress surrounding mesial implants followed by three-piece and one-piece frameworks.

**TABLE 9:** Site of maximum von Mises stresses on the implant in the right unilateral molar clenching.

	Buccal		Lingual	
	Balanced side	Functional side	Balanced side	Functional side
One-piece	+			+
Two-piece		+		+
Three-piece	+		+	

‘+’ refers to the site of maximum stress on the implant from the buccal/lingual view in different segmental frames.

## Discussion

In the treatment of edentulous jaws, MF is known to be associated with restoration failure and peri-implant bone resorption (Burch and Borchers, 1970; Goodkind and Heringlake, 1973; Hylander, 1984; Abdel-Latif et al., 2000; Law et al., 2012; Müller et al., 2012; Sivaraman et al., 2016) which varies with the displacement of different facial types (Prasad et al., 2013). In this study, we employed FEM to investigate the effects of MF on stress distribution in the seven designs of implant-supported fixed prostheses of three facial types. Despite the differences in the mandibular morphology of patients with different facial types (Gates and Nicholls, 1981; Canabarro and Shinkai, 2006; Sivaraman et al., 2016), this discrepancy did not affect MF (Suresh, 2005), and there were no differences in the mandibular incisors’ inclination and symphyseal area of three facial types, and the chin width differed only slightly among females but not among males (Sella Tunis et al., 2021). Therefore, the same arch morphology for different facial types was applied in this study. Also, different cohesive forces applied at the bilateral condyles were used to mimic the muscle strength of different facial types through FEA. Moreover, seven models, which have been widely used in clinical practice, were used to investigate the effects of MF on stress distribution in both the mandible and the prosthesis. The stress in the denture in the present study was below 600 MPa, which is lower than that of the maximum stress that implants can withstand; hence, the model settings were rational (Zarone et al., 2003). The results aimed to provide clinical guidelines on the design and application of implant superstructure.

The primary aim of implant-supported fixed restorations is to achieve optimal biomechanical distribution both at the level of the prosthetic superstructure and implant (Martin-Fernandez et al., 2018). In this study, the elastic flexion of the mandible is limited by the presence of a full-arch structure that rigidly connects the implants (0.47 and 0.57 in models 1, 4, and 7, respectively), while the division of the framework into separate bridges restores more natural flexibility (over 0.85) (Fischman, 1976; Fischman, 1990; Hobkirk and Havthoulas, 1998). The results are supportive of the theory that inflexible full-arch prostheses can provide additional resistance, thus counteracting the effects of MF when there is a single unilateral posterior framework (Yokoyama et al., 2005; Naini and Nokar, 2009). Indeed, in this study, the stresses on the restoration and the mandible were more uniform in the one-piece framework, and no high-stress regions were observed. In contrast, in the case of the segmented framework, higher stress was observed in the loading conditions of opening and unilateral molar clenching in both mesial implants and the superstructure (as shown in Figure 8; Table 8). Therefore, the one-piece framework exhibited the best biomechanical environment in both mandibular opening and occlusal movement. Given this fact, a one-stage framework should be taken into account when planning implant-supported restorations, especially for the patient with high masticatory muscle strength and a large elastic flexion of the mandible (Nokar and Baghai Naini, 2010).

However, it is still controversial whether such a one-piece framework, which distributes stresses uniformly by limiting physiological bending, is optimal. There have been case reports on the recovery of pain and symptoms in patients by dividing the prosthesis into several sections (de Oliveira and Emtiaz, 2000; Paez et al., 2003). We think this may be related to the possibility of misalignment between the prosthesis and the implant position. In a segmented superstructure, it will be easier to perform small adjustments (das Neves et al., 2012; Natali et al., 2006), thus making it easier to achieve passive seating, rather than reduced stress on the restoration and mandible. In addition, others advocated that segmented superstructures did not restrict the physiological bending of the mandible and could reduce stress at the mid-line of the superstructure (Fischman, 1976; Fischman, 1990; Paez et al., 2003; Martin-Fernandez et al., 2018). However, as Modi et al. (2015) concluded that although nonrigid connectors lead to a decrease in stress at the level of the prosthesis, they lead to an increase in stress at the level of the alveolar ridge. Moreover, the most common complications in the use of multi-unit abutments are abutment screw loosening (Freitas et al., 2012; Rocha Ferreira et al., 2018; Benalcázar Jalkh et al., 2021; Sánchez-Torres et al., 2021). Just as the conclusion obtained in our study, stress was mostly significantly concentrated at the joint between the abutments and segmental superstructure, which would increase the significant incidence of mechanical problems.

Additionally, in these FEAs (Hobkirk and Havthoulas, 1998; Zarone et al., 2003), the loading forces applied to the bilateral condyles were mostly 10–16 N, comparable to those of the dolichofacial type in this study, in which the stresses around the implants and in the jawbone were relatively low with no significant stress concentration. Nonetheless, in other facial types, especially the brachyfacial type, greater masticatory muscle strength and MF resulted in significant stress around the anterior implants and at the connection between abutments and the superstructure in the segmented framework, which could easily lead to the failure and abscission of the superstructure. It suggests the importance of optimizing the design of edentulous fixed implant restorations according to the facial types. According to the results, for the brachyfacial type, a one-piece framework should be chosen to be more conducive to the long-term preservation of the restoration. Regarding the mesofacial and dolichofacial types, a one-piece framework is also recommended for the patient with a large occlusal force or has oral para-functions such as bruxism, which means having greater MF. If the patient has poor oral hygiene or is a smoker, a segmented design is more suitable because cleaning is more easily. In this case, it is also important to achieve the passive fit of the segmental restoration to improve the strength of the joint.

In addition to facial type, we also attempted to explore the relationship between sex and MF. In the field of forensic medicine, the accuracy of mandibular ramus flexure for gender judgment ranges from 50 to 80% (Koski, 1996). However, some studies have also concluded that gender cannot be one of the influencing factors of MF (Oettlé et al., 2005; Lin et al., 2014; Wolf et al., 2019; Gülsoy et al., 2022). In our study, by comparing the stress environment between different genders of the same facial type, the difference in the maximum stress on the jaw was only within 10 MPa, and the difference in the maximum stress on the restorations also did not exceed 30 MPa, with no significance for the design of implant-fixed prostheses.

The number and sites of implants (Flanagan, 2005) and the material of the superstructure (Favot et al., 2014) also affected the stress on the jaw and prosthesis. Comparing models 1 and 4 with model 7, it can be seen that the implant-supported fixed denture with six implants and without any cantilever has a more excellent biomechanical environment and the maximum stress values on the neck of implants (36 and 46), and the superstructures in models 1 and 4 are all smaller than those (35 and 45) in model 7, so avoiding or reducing cantilever length of the superstructure can be more conducive to the stress dispersion (McCartney, 1992). Moreover, in any framework, stress values of prostheses and mesial implants increased as the distal implants were more distally located. This is mainly because the physiological bending of the mandible occurs in the distal segment of the mandibular foramen (Sivaraman et al., 2016), and there is more span in the anterior segment of the superstructure as the distal implant position is remote, which is more detrimental to the preservation of the bone in the anterior segment of the mandible. Regarding the material of the superstructure, titanium was widely used for edentulous fixed implant frameworks due to its similar elastic modulus to the bone. In line with Favot et al. (2014), the stresses on the restorations of all seven models of the titanium group were lower than those of the zirconia group. This indicated that when subjected to the same stress, the titanium framework was more adapted to the physiological bending of the mandible than the zirconia framework, thus leading to fewer mechanical complications.

Nonetheless, there are still some limitations to this study. For the experimental method of three-dimensional finite element, it is not possible for a mathematical/computational model to reproduce all biological characteristics as exactly as possible, and the FEA must simplify the design of materials, loading, and boundary conditions (Martin-Fernandez et al., 2018). First of all, the superstructure was segmented as 0.5 mm apart from each other, with no contact and no friction between each other. Second, the friction between the implant, central screw and abutment was ignored in this experiment. Considering the aforementioned simplifications and assumptions, the distributional data of this study should be read from a qualitative point of view, instead of a quantitative one, and is only a treatment of the clinical situation.

## Conclusion

MF and designs of the implant-supported fixed prostheses of the edentulous mandible are the key factors affecting the stress distribution of the mandibular prosthesis, and the influence degree is different for people with different facial types.

·In the treatment of edentulous jaws, the number of implants should be increased if the positioning of fixtures in the posterior regions is feasible, from an anatomical and surgical point of view, to avoid cantilever and excessive spacing between implants. Choosing a material with a smaller modulus of elasticity for the framework is recommended.

·For the brachyfacial type, it is recommended to choose the one-piece superstructure across the dental arch. For the mesofacial type and the dolichofacial type, it can be selected according to the actual situation. If the patient has a large bite force and oral side function, it is recommended to choose the integrated structure across the dental arch.

## Data Availability

The original contributions presented in the study are included in the article/Supplementary Meterial, further inquiries can be directed to the corresponding authors.
